# Clinical Heterogeneity in two patients with Noonan-like Syndrome associated with the same SHOC2 mutation

**DOI:** 10.1186/1824-7288-38-48

**Published:** 2012-09-20

**Authors:** Donatella Capalbo, Maria Giuseppa Scala, Daniela Melis, Giorgia Minopoli, Nicola Improda, Loredana Palamaro, Claudio Pignata, Mariacarolina Salerno

**Affiliations:** 1 Department of Pediatrics, “Federico II” University of Naples, Naples, Italy

**Keywords:** Noonan-like Syndrome with loose anagen hair, Growth hormone deficiency, Growth hormone insensitivity

## Abstract

Noonan-like syndrome with loose anagen hair (NS/LAH; OMIM #607721) has been recently related to the invariant c.4A > G missense change in *SHOC2*. It is characterized by features reminiscent of Noonan syndrome. Ectodermal involvement, short stature associated to growth hormone (GH) deficiency (GHD), and cognitive deficits are common features. We compare in two patients with molecularly confirmed NS/LAH diagnosis, the clinical phenotype and pathogenetic mechanism underlying short stature. In particular, while both the patients exhibited a severe short stature, GH/IGFI axis functional evaluation revealed a different pathogenetic alteration, suggesting in one patient an upstream alteration (typical GHD) and in the other one a peripheral GH insensitivity.

Since only a few cases of NS/LAH associated to SHOC2 mutations have been so far described, the complex phenotype of the syndrome and the exact mechanism impairing GH/IGFI axis still remain to be elucidated and studies on larger cohort of subjects are needed to better delineate this syndrome.

## Background

“Noonan-like syndrome with loose anagen hair” (NS/ LAH OMIM60772) is a syndrome first described in 2003, characterized by features reminiscent of NS and a unique pattern of ectodermal abnormalities [1]. Recently, Cordeddu et al. discovered that an invariant mutation in SHOC2, c.4A > G, predicting the p.Ser2Gly change in the encoded protein, underlies this condition. SHOC2 encodes a scaffold protein that positively modulates RAS-MAPK signal flow. The mutation was documented to promote N-myristoylation of the protein, and to drive aberrant targeting of SHOC2 to the plasma membrane and increased ERK activation in a cell context-specific fashion [2]. The phenotype of these subjects is characterized by facial features reminiscent of NS, short stature, cognitive deficits, distinctive hyperactive behavior, congenital hearth disease, and easily pluckable, sparse, thin, slow-growing hair in the anagen phase but lacking an inner and outer root sheaths.

Short stature is one of the most common features observed in NS as well as in other disorders associated to RAS-MAPK dysregulation. It is well known that the RAS-MAPK transduction pathway plays a key role in growth hormone (GH) signaling [3]. However, the exact mechanism of impaired GH-IGF1-axis in patients with aberrant RAS-MAPK signaling is still controversial. An altered response of GH to stimulation tests has been reported in some children [4,5], whereas other authors have reported normal GH secretion with low IGF1 levels suggesting a peripheral GH insensitivity (GHI) [6,7]. GHI is a condition characterized by the peripheral resistance to GH action [8-10]. Transduction of the signals elicited by GHR is mediated by the JAK/STAT pathway. Tyrosine phosphorylation of JAK2 and STAT5 plays a crucial role in such a process, which eventually results in gene transcription [11-13]. The most common form of GHI is due to GHR mutations; other forms are caused by abnormalities in the signaling cascade downstream to GHR and, in particular, involving STAT5b [9,11]. In subjects with STAT5b defects short stature is associated with immunodeficiency and severe pulmonary involvement [8,14,15].

In children with NS/LAH short stature is mainly associated to proven GH deficiency (GHD) [2].

So far, only a few patients with NS/LAH have been described and the phenotype of the syndrome still remains to be delineated. We recently reported on a patient affected with this syndrome, who exhibited a GHI [16]. Herein, we compare the clinical phenotype and the pathogenetic mechanism underlying short stature and abnormal GH/IGFI axis between the already described patient and a further affected child with the same mutation of the SHOC2 gene.

### Subjects

#### Patient 1

**Figure 1:**
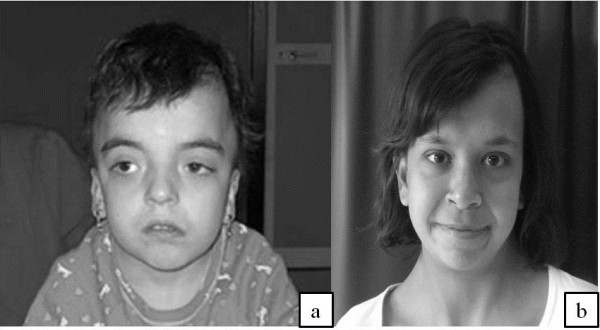
Patient 1 at 9 years of age (a) and patient 2 at 13 years (b) in a frontal view.

The patient was born preterm (34 weeks of gestation) with a pregnancy complicated by polyhydramnios. Parents were not consanguineous. Birth weight was 2.450 kg (75^th^ centile), length was 45 cm (25^th^-50^th^ centile). She was referred to our attention at the age of 7 years for short stature. Physical examination revealed macrocephaly, high forehead, epicanthic folds, palpebral ptosis, hyperthelorism, high and narrow palate, pterigium colli, pectus excavatum with widely spaced nipples, deep palmar and plantar creases (Figure 1a). She had severe growth delay: weight was 13 kg (−4.4 SDS) and height was 97 cm (−5.7 SDS), with impaired linear growth velocity (3 cm/year). Bone age was delayed by three years. Karyotype was normal (46, XX). Neurological evaluation revealed a mild psychomotor delay (IQ: 52) with impairment of verbal and language abilities. Cerebral MRI showed hypoplastic corpus callosum. Based on these features, NS disease genes were screened (*PTPN11*, *KRAS*, *SOS1*, *MEK1*, *BRAF*) but mutation analysis failed to reveal any causative mutation.

Biochemical evaluation of GH-IGF1 axis, performed to investigate the cause underlying short stature, revealed low serum levels of IGF1 (27 ng/ml, nv 60–350) and normal GH peak after arginine stimulation (16 ng/ml), thus excluding a GHD [17] and suggesting a condition of mild GH insensitivity (GHI). An IGFI generation test confirmed the condition of GHI, revealing only a mild increase of IGFI levels (55 ng/ml) after stimulation with exogenous GH with respect to basal values (30 ng/ml). Moreover, the peak value obtained was still below the mean value for age (60–350 ng/ml). GHI was not related to mutations in GH receptor in that molecular analysis of GHR gene was normal. Moreover, intracellular signalling elicited by GHR perturbation, evaluated through the analysis of GH-induced tyrosine phosphorylation of Signal Transducers and Activators of Transcription (STAT)5, was normal.

**Figure 2:**
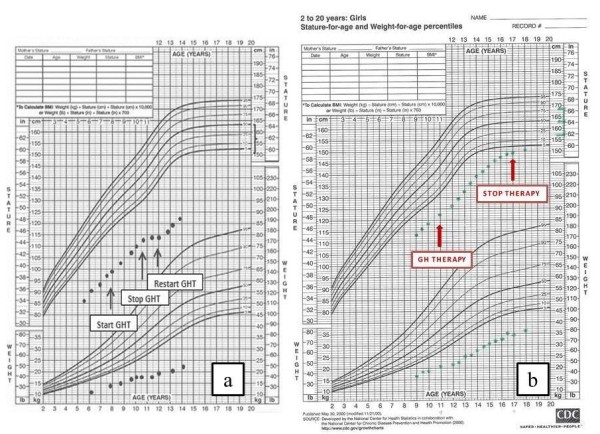
Longitudinal growth curve of the 2 patients before and after (arrow) growth hormone (GH) therapy.

The patient underwent a therapeutic trial with recombinant human (rh) GH at a mean dose of 45 μg/kg/die. However, no significant improvement in linear growth was observed. Nevertheless, an arrest of growth velocity was observed when GH treatment was stopped. Thus, GH was re-started in order to normalize growth velocity (Figure 2a).

Over time, several ectodermal abnormalities became evident, including nail dystrophy, dry, fine textured, sparse hair, which were easily pulled from the scalp, and dry, hairless, darkly pigmented skin. Such a distinctive association prompted SHOC2 mutation analysis which confirmed the presence of the disease-causing mutation (c.4A > G, p.Ser2Gly), thus confirming diagnosis of NS/LAH. At 9 years, she suddenly presented with complex motor and vocal tics characterized by repetitive stereotyped movements predominantly involving facial and shoulder muscles and repetitive sounds, which persisted for more than 1 year. Major causes of secondary tics disorders in childhood were excluded, including occurrence of neurodegenerative disease, central nervous system infections, pediatric autoimmune neuropsychiatric disorder associated with streptococcal infection (PANDAS) or drug-related effects [18]. A few months later, she presented with a generalized convulsive event. Electroencephalography revealed the presence of focal abnormalities in both parietal and occipital areas, whereas MRI confirmed the previous finding of corpus callosum hypoplasia and showed diffuse reduction in the white matter. She was then treated with carbamazepine with a good control on seizures but without any effect on tic disorders. She is now 13 years old. Despite a 6-years GH treatment, she still has a severe short stature (height −5 SDS) (Figure 2a), and IGF1 levels well below the normal range (93.5 ng/ml; nv 180–780) [16].

#### Patient 2

The patient was born at term (40 weeks of gestation) after a normal pregnancy and caesarian delivery from not consanguineous parents. Birth weight was 3.75 kg (50-75^th^ centile), length was 48 cm (10-25^th^ centile) and head circumference 37 cm (>90°). At birth, a large atrial septal defect with pulmonary hyperflow was detected.

She was referred to our Department at the age of 9 years because of short stature. Physical examination revealed macrocephaly, high forehead, epicanthic folds, low-set and posterior angulated left ear, hyperthelorism, short neck with redundant skin, pectus excavatum, fine, dry, fine textured, sparse hair, darkly pigmented skin with cafe au lait spots, tuberous angioma in the left hypochondrium, hyperextensible joints and mild scoliosis (Figure 1b). She had a severe growth delay: weight was 16.5 kg (−3.72 SDS) and height was 113.5 cm (−3.21 SDS). Bone age was delayed by two years. Karyotype was normal (46, XX). She had only a mild psychomotor delay (IQ 65) and behavioral problems. Cerebral MRI showed type I Chiari malformation.

The biochemical evaluation showed a subclinical hypothyroidism (SH) on the basis of a mild increase of TSH levels in the absence of thyroid autoantibodies which normalized over time, associated with normal values of circulating hormones, thus not requiring replacement therapy [19-22]. The presence of APA was also excluded as an autoimmune cause of short stature [23]. Biochemical evaluation of GH-IGF1 axis, performed to investigate the cause of the short stature, revealed abnormal GH peak after clonidine stimulation (9 ng/ml) and arginine stimulation (5.3 ng/dL) with serum levels of IGF1 at the lower limit of normal range (70.4 ng/ml ), indicative of a condition of partial GH deficiency She was then started on recombinant human (rh) GH at a mean dose of 40 μg/kg/die, with only a blunted response to treatment (Figure 2b). At the age of 17 years she stopped the therapy (Figure 2b).

Later on during the follow-up, the girl showed an improvement in her psychomotor development and behavior skills.

Since at the age of 14 years no signs of pubertal development were evident, a LHRH test was performed revealing normal activation of the gonadotropic axis (peak of LH 14.4 mU/mL; peak of FSH 21.7 mU/mL). Nevertheless, no pubertal progression was observed in the following years and at the age of 16 years she was started on hormonal replacement.

The complex clinical phenotype prompted to the clinical diagnosis of NS/LAH and molecular analysis of *SHOC2* revealed the presence of the disease-causing mutation (c.4A > G, p.Ser2Gly).

In Table 1 a comparison of the two phenotypes has been reported.

**Table 1:** Phenotipic features of Noonan-like syndrome with loose anagen hair in our two patients

	**Patient 1**	**Patient 2**
Skin and hair		
Dark skin	+	+
Hyperkeratosis	-	-
Dermatitis/eczema	-	-
Sparse/absent scalp hair	+	+
Curly hair	-	-
Loose anagen hair	+	+
Head and neck		
Macrocephaly	+	+
Eyes		
Ptosis	-	+
Epicanthal folds	+	+
Nose		
Depressed nasal bridge	-	-
Anteverted nostrils	-	-
Oral cavity		
High-arched palate	-	+
Ears		
Apparently low-set ears	+	-
Retroverted ears Neck	+	-
Neck		
Broad, short, webbed	+	+
Chest		
Pectus excavatum	+	+
Cardiovascular		
VSD/ASD/PDA	+	-
Cerebral abnormalities		
Dilated ventricles	-	-
Mental retardation	-	+
Tic	-	+
Growth and development		
Short stature	+	+
Lymphatic dysplasias	+	-

## Discussion

We reported on two patients with molecularly confirmed diagnosis of NS/LAH exhibiting different features of the disease and different pathogenetic mechanisms underlying the impairment of GH-IGFI axis resulting in short stature.

Both patients had the similar facial dysmorphism, skeletal abnormalities, ectodermal abnormalities and psychomotor delay, even though these features were more pronounced in the patient 1. Patient 1 also had epilepsy and atypical neurologic signs such as chronic vocal and motor tics, never previously reported in patients with *SHOC2* mutations or in any other condition due to RAS-MAPK dysregulation. The precise etiology of chronic tics in childhood often remains elusive. However, abnormalities in the dopamine pathways have been supposed as possible mechanism underlying neurotransmitter alterations [24]. Recent evidence suggests that RAS-MAPK pathway is involved in signal transduction that follows dopamine receptors triggering [25]. Thus, one could speculate that in our patient dysregulation of RAS-MAPK due to dysregulated SHOC2 function might be related to tics disorders through an alteration of dopamine-signaling. In keeping with this, studies on Gilles de la Tourette syndrome (GTS), one of the most frequent causes of chronic tics in infancy, also indicate a potential role of immune activation, mediated by RAS-MAPK stimulating cytokines, in the pathogenesis of tics and other hyperkinetic disorders [26].

As for developmental and behavioral features, again patient 1exhibited persistent alterations during adolescence, differently from patient 2 who only had a mild delay improving over time.

Both patients had severe short stature, even though GH/IGFI axis was differently altered. In fact, in the patient 1 the evaluation of GH-IGFI axis revealed a condition of GHI, whereas in patient 2 a partial GH deficiency was found. So far, GHD represents the most common cause of short stature in NS/LAH syndrome. Differently, GHI has never been described in NS/LAH subjects. In our second patient, molecular analysis of GHR and GH-induced phosphorylation of STAT5 were normal, thus excluding the most common receptorial or postreceprtorial causes of peripheral insensitivity to GH. Since both patients with GHD and with GHI share the same mutation, it seems unlikely that this mutation cause GHI per se. However, it is conceivable that SHOC2 mutation alters GHR signaling through dysregulation of RAS-MAPK pathway, thus contributing to cause GHI. In keeping with this, GH replacement therapy was unsuccessful in the patient 1. The observation that also in the patient 2, in spite of GHD the GH replacement therapy was not fully successful indicates that *SHOC2* has multiple roles in either central or peripheral control of GH-IGFI axis functionality.

The clinical heterogeneity in our 2 patients carrying the same mutation, is not surprising as other monogenic diseases have been already shown to have a different phenotype despite the same causing mutation [27-30]. For most of these conditions, molecular basis of this heterogeneity are not well delineated. Disease modifying genes, variation in environmental exposures, as well as system dynamics may come into play in modulating clinical expression of the disease [31].

## Conclusion

In conclusion, we reported on two patients with NS/LAH due to the invariant c.4A > G mutation in *SHOC2*, with a different phenotype of the disease and severe short stature related to different mechanisms impairing GH-IGFI axis. Since only a few cases of the NS/LAH have so far been described, studies on larger cohort of subjects are needed to better delineate the phenotypic heterogeneity and to better define the pathogenetic mechanism underlying short stature in these patients.

### Consent

Written informed consent was obtained from the parents of the patients for publication of this Case report and any accompanying images. A copy of the written consent is available for review by the Editor-in-Chief of this journal.

## Competing interests

The authors declare that they have no competing interests.

## Authors’ contributions

All authors have equally participated in drafting of the manuscript and/or critical revision of the manuscript for important intellectual content. All authors read and approved the final manuscript.

## References

[B1] Mazzanti L Cacciari E Cicognani A Bergamaschi R Scarano E Forabosco A Noonan-like syndrome with loose anagen hair: a new syndrome? Am J Med Genet 2003 118A 279 286 10.1002/ajmg.a.10923 12673660

[B2] Coreddu V Di Schiavi E Pennacchio LA Ma’ayan A Sarkozy A Fodale V Cecchetti S Cardinale A Martin J Schackwitz W Lipzen A Zampino G Mazzanti L Diglio MC Martinelli S Flex E Lepri F Bartholdi D Kutsche K Ferrero GB Anichini C Selicorni A Rossi C Tenconi R Zenker M Merlo D Dallapiccola B Iyengar R Bazzicalupo P Gelb BD Tartarglia M Mutation of SHOC2 promotes aberrant protein N-myristoilation and causes Noonan-like syndrome with loose anagen hair Nat Genet 2009 41 1022 1026 10.1038/ng.425 19684605 PMC2765465

[B3] Padidela R Camacho Hubner C Attie KM Savage MO Abnormal growth in Noonan syndrome: genetic and endocrine features and optimal treatment Horm Res 2008 70 129 136 10.1159/000145016 18663312

[B4] Romano AA Blethen SL Dana K Noto RA Growth hormone treatment in Noonan syndrome: the national cooperative growth study experience J Pediatr 1996 128 S18 S21 10.1016/S0022-3476(96)70005-7 8627463

[B5] Cotteril AM McKenna WJ Brady AF Sharland M Elsawi M Yamada M Camacho-Hubner C Kelnar CJ Dunger DB Patton MA Savage MO The short term effects of growth hormone therapy on height velocity and cardiac ventricular wall thickness in children with Noonan’s syndrome J Clin Endocrinol Metab 1996 81 2291 2297 10.1210/jc.81.6.2291 8964866

[B6] Limal JM Parfait B Cabrol S Bonnet D Leheup B Lyonnet S Vidaud M Le Bouc Y Noonan syndrome: relationship between genotype, growth and growth factors J Clin Endocrinol Metab 2006 91 300 306 10.1210/jc.2005-0983 16263833

[B7] Ferreira LV Souza SA Arnhold IJ Mendoca BB Jorge AA PTPN11 (protein tyrosine phosphatase, nonreceptor type 11) mutations and response to growth hormone therapy in children with Noonan syndrome J Clin Endocrinol Metab 2005 90 5377 5381 10.1210/jc.2005-0995 15956085

[B8] Rosenfeld RG Rosenbloom AL Guevara-Aguirre J Growth hormone (GH) insensitivity due to primary GH receptor deficiency Endocr Rev 1994 15 369 390 8076588 10.1210/edrv-15-3-369

[B9] Salerno M Balestrieri B Matrecano E Officioso A Rosenfeld RG Di Maio S Fimiani G Ursini MV Pignata C Abnormal GH receptor signaling in children with idiopathic short stature J Clin Endocrinol Metab 2001 86 3882 3888 10.1210/jc.86.8.3882 11502828

[B10] Ursini MV Gaetaniello L Ambrosio R Matrecano E Apicella AJ Salerno MC Pignata C Atypical X-linked SCID phenotype associated with growth hormone hyporesponsiveness Clin Exp Immunol 2002 129 3 502 509 10.1046/j.1365-2249.2002.01823.x 12197892 PMC1906458

[B11] Hwa V Nadeau K Wit JM Rosenfeld RG STAT5b deficiency: lessons from STAT5b gene mutations Best Pract Res Clin Endocrinol Metab 2011 25 61 75 10.1016/j.beem.2010.09.003 21396575

[B12] Amorosi S Russo I Amodio G Garbi C Vitiello L Palamaro L Adriani M Vigliano I Pignata C The cellular amount of the common gamma-chain influences spontaneous or induced cell proliferation J Immunol 2009 182 5 3304 3309 10.4049/jimmunol.0802400 19234229

[B13] Vigliano I Palamaro L Bianchino G Fusco A Vitiello L Grieco V Romano R Salvatore M Pignata C Role of the common γ chain in cell cycle progression of human malignant cell lines Int Immunol 2012 24 159 167 10.1093/intimm/dxr114 22223761

[B14] Montella S Maglione M Bruzzese D Mollica C Pignata C Aloj G Manna A Esposito A Mirra V Santamaria F Magnetic resonance imaging is an accurate and reliable method to evaluate non-cystic fibrosis paediatric lung disease Respirology 2012 17 1 87 91 10.1111/j.1440-1843.2011.02067.x 21943039

[B15] Montella S Santamaria F Salvatore M Pignata C Maglione M Iacotucci P Mollica C Assessment of chest high-field magnetic resonance imaging in children and young adults with noncystic fibrosis chronic lung disease: comparison to high-resolution computed tomography and correlation with pulmonary function Invest Radiol 2009 44 9 532 538 10.1097/RLI.0b013e3181b4c1ba 19652613

[B16] Capalbo D Melis D De Martino L Palamaro L Riccomagno S Bona G Cordeddu V Pignata C Salerno M Noonan-like syndrome with loose anagen hair associated with growth hormone insensitivity and atypical neurological manifestations Am J Med Genet A 2012 158A 4 856 860 10.1002/ajmg.a.35234 22419608

[B17] Corneli G Di Somma C Prodam F Bellone J Bellone S Gasco V Baldelli R Rovere S Schneider HJ Gargantini L Gastaldi R Ghizzoni L Valle D Salerno M Colao A Bona G Ghigo E Maghnie M Aimaretti G Cut-off limits of the GH response to GHRH plus arginine test and IGF-I levels for the diagnosis of GH deficiency in late adolescents and young adults Eur J Endocrinol 2007 157 6 701 708 10.1530/EJE-07-0384 18057376

[B18] Dooley JM Tic Disorders in childhood Semin Pediatr Neurol 2006 13 231 242 10.1016/j.spen.2006.09.004 17178353

[B19] Wasniewska M Salerno M Cassio A Corrias A Aversa T Zirilli G Capalbo D Bal M Mussa A De Luca F Prospective evaluation of the natural course of idiopathic subclinical hypothyroidism in childhood and adolescence Eur J Endocrinol 2009 160 3 417 421 19074464 10.1530/EJE-08-0625

[B20] Wasniewska M Corrias A Aversa T Valenzise M Mussa A De Martino L Lombardo F De Luca F Salerno M Comparative evaluation of therapy with L-thyroxine versus no treatment in children with idiopathic and mild subclinical hypothyroidism Horm Res Paediatr 2012 77 6 376 381 10.1159/000339156 22699818

[B21] Cerbone M Bravaccio C Capalbo D Polizzi M Wasniewska M Cioffi D Improda N Valenzise M Bruzzese D De Luca F Salerno M Linear growth and intellectual outcome in children with long-term idiopathic subclinical hypothyroidism Eur J Endocrinol 2011 164 4 591 597 10.1530/EJE-10-0979 21292920

[B22] Radetti G Maselli M Buzi F Corrias A Mussa A Cambiaso P Salerno M Cappa M Baiocchi M Gastaldi R Minerba L Loche S The natural history of the normal/mild elevated TSH serum levels in children and adolescents with Hashimoto's thyroiditis and isolated hyperthyrotropinaemia: a 3-year follow-up Clin Endocrinol (Oxf) 2012 76 3 394 398 10.1111/j.1365-2265.2011.04251.x 21981142

[B23] De Bellis A Salerno M Conte M Coronella C Tirelli G Battaglia M Esposito V Ruocco G Bellastella G Bizzarro A Bellastella A Antipituitary antibodies recognizing growth hormone (GH)-producing cells in children with idiopathic GH deficiency and in children with idiopathic short stature J Clin Endocrinol Metab 2006 91 7 2484 2489 10.1210/jc.2006-0040 16621907

[B24] Du JC Chiu TF Lee KM Wu H Tourette Syndrome in children: an update review Pediatr Neonatol 2010 51 255 264 10.1016/S1875-9572(10)60050-2 20951354

[B25] Zhen X Zhang J Jhonson GP Friedman E D4 dopamine receptor differentially regulates Akt/Nuclear factor-Kb and extracellular signal-regulated kinase pathways in D4MN9D cells Mol Pharmacol 2010 60 857 864 11562449

[B26] Martino D Dale RC Gilbert DL Giovannoni G Leckman JF Immunopathogentic mechanisms in Tourette syndrome: a critical review Mov Disord 2009 24 1267 1279 10.1002/mds.22504 19353683 PMC3972005

[B27] Capalbo D Fusco A Aloj G Improda N Vitiello L Dianzani U Betterle C Salerno M Pignata C High intrafamilial variability in autoimmune polyendocrinopathy-candidiasis-ectodermal dystrophy: a case study J Endocrinol Invest 2012 35 1 77 81 22071465 10.3275/8055

[B28] Capalbo D Mazza C Giordano R Improda N Arvat E Cervato S Morlin L Pignata C Betterle C Salerno M Molecular background and genotype-phenotype correlation in autoimmune-polyendocrinopathy-candidiasis-ectodermal-distrophy patients from Campania and in their relatives J Endocrinol Invest 2012 35 2 169 173 21508664 10.3275/7677

[B29] Mazza C Buzi F Ortolani F Vitali A Notarangelo LD Weber G Bacchetta R Soresina A Lougaris V Greggio NA Taddio A Pasic S de Vroede M Pac M Kilic SS Ozden S Rusconi R Martino S Capalbo D Salerno M Pignata C Radetti G Maggiore G Plebani A Notarangelo LD Badolato R Clinical heterogeneity and diagnostic delay of autoimmune polyendocrinopathy-candidiasis-ectodermal dystrophy syndrome Clin Immunol 2011 139 1 6 11 10.1016/j.clim.2010.12.021 21295522

[B30] Capalbo D Giardino G De Martino L Palamaro L Romano R Gallo V Cirillo E Salerno M Pignata C Genetic basis of altered central tolerance and autoimmune diseases: a lesson prom AIRE mutations Int Rev Immunol 2012 in press 10.3109/08830185.2012.697230 23083345

[B31] Ming JE Muenke M Multiple hits during early embryonic development: digenic diseases and holoprosencephaly Am J Hum Genet 2002 71 1017 32 10.1086/344412 12395298 PMC385082

